# Leucine supplementation differentially enhances pancreatic cancer growth in lean and overweight mice

**DOI:** 10.1186/2049-3002-2-6

**Published:** 2014-03-31

**Authors:** Kristyn A Liu, Laura M Lashinger, Audrey J Rasmussen, Stephen D Hursting

**Affiliations:** 1 Department of Nutritional Sciences, University of Texas at Austin, Austin, TX 78723, USA; 2 Department of Molecular Carcinogenesis, University of Texas M.D. Anderson Cancer Center, 1808 Park Road 1c, Smithville, TX 78957, USA

**Keywords:** Pancreatic cancer, Leucine supplementation, Calorie restriction, mTOR, Glucose metabolism

## Abstract

**Background:**

The risk of pancreatic cancer, the 4th deadliest cancer for both men and women in the United States, is increased by obesity. Calorie restriction (CR) is a well-known dietary regimen that prevents or reverses obesity and suppresses tumorigenesis in a variety of animal models, at least in part via inhibition of mammalian target of rapamycin (mTOR) signaling. Branched-chain amino acids (BCAA), especially leucine, activate mTOR and enhance growth and proliferation of myocytes and epithelial cells, which is why leucine is a popular supplement among athletes. Leucine is also increasingly being used as a treatment for pancreatic cancer cachexia, but the effects of leucine supplementation on pancreatic tumor growth have not been elucidated.

**Results:**

Supplementation with leucine increased pancreatic tumor growth in both lean (104 ± 17 mm^3^ versus 46 ± 13 mm^3^; *P* <0.05) and overweight (367 ± 45 mm^3^ versus 230 ± 39 mm^3^; *P* <0.01) mice, but tumor enhancement was associated with different biological outcomes depending on the diet. In the lean mice, leucine increased phosphorylation of mTOR and downstream effector S6 ribosomal protein, but in the overweight mice, leucine reduced glucose clearance and thus increased the amount of circulating glucose available to the tumor.

**Conclusions:**

These findings show that leucine supplementation enhances tumor growth in both lean and overweight mice through diet-dependent effects in a murine model of pancreatic cancer, suggesting caution against the clinical use of leucine supplementation for the purposes of skeletal muscle enhancement in cachectic patients.

## Background

Effective prevention and treatment strategies are urgently needed for pancreatic cancer, the 4th leading cause of cancer-related death in both men and women in the United States [1]. Less than 15% of pancreatic cancer patients have localized disease amenable to curative resection, and the overall 5-year survival rate in affected patients is less than 5% [2]. Obesity is an established pancreatic cancer risk and progression factor in humans and animal models [3-5]. In contrast, calorie restriction (CR) prevents or reverses obesity and related metabolic perturbations and pancreatic tumor development and/or progression in experimental models [6-11]; the impact of CR on human pancreatic cancer has not been well studied. CR results in a negative energy balance state and exerts its antitumor effects, at least in part, through decreased mammalian target of rapamycin (mTOR) signaling in many epithelial tissues [7-9,11,12]. mTOR acts as a nutrient sensor that regulates protein synthesis, cell survival, and proliferation in response to growth factor levels, nutrient availability, and intracellular energy status. We have previously established that rapamycin (a selective mTOR inhibitor), and metformin (an indirect inhibitor of mTOR signaling through its effects on gluconeogenesis and associated activation of AMPK-regulated signals), partially mimic the tumor inhibitory effects of CR on transplanted pancreatic tumor growth [13].

Branched-chain amino acids (BCAAs), which account for over 20% of total dietary protein intake, are known activators of the mTOR pathway in muscle and epithelial tissues [14-16]. Of the three BCAAs, leucine exerts the most potent effect on mTOR activation and enhancement of protein synthesis in various tissues, including skeletal muscle [17,18]. Athletes commonly use leucine supplementation to activate mTOR-regulated protein synthesis and accelerate muscle repair and regeneration after injuries or intense bouts of exercise [19]. Leucine supplementation is also increasingly being recommended to reduce the muscle wasting that occurs with cancer cachexia [20]. Cachexia is characterized by involuntary weight loss and muscle wasting, is associated with increased morbidity and mortality, and frequently occurs in pancreatic cancer patients [21]. Increased muscle protein synthesis in response to leucine-induced mTOR activation has been shown to inhibit muscle wasting in mouse models of cancer cachexia and in cancer patients [22-25]. However, the rates of protein synthesis increase to a much greater extent in tumors than in muscle [24], suggesting that while leucine supplementation may protect against cancer-associated cachexia, it may also enhance the progression of the cancer.

Unfortunately, studies of the effects of leucine supplementation on cancer are limited. Long-term leucine supplementation (2% of diet, w/w) promoted bladder cancer development in rats treated with a known bladder carcinogen [26,27], but no studies have connected leucine supplementation with tumor growth. In the present study, we tested the effect of leucine supplementation on transplanted Panc02 mouse pancreatic cancer growth and mTOR signaling in the context of lean mice (fed a CR diet regimen) or overweight mice (fed a high calorie control diet regimen). Our findings suggest that leucine enhances pancreatic tumor progression in lean and overweight mice, and the underlying mechanisms may differ by weight status.

## Methods

### Mice and dietary interventions

All experiments were conducted under a protocol approved by the Institutional Animal Care and Use Committee at the University of Texas at Austin. Eighty-eight male C57BL/6 mice were obtained from Charles River Breeding Laboratories (Wilmington, MA, USA) at 6 to 8 weeks of age, and upon arrival were singly-housed in a semibarrier facility at the University of Texas at Austin Animal Resource Center and fed a chow diet during a one-week acclimation period. Mice were then randomized to receive one of four diets for 27 weeks: (i) AIN-76A control diet consumed *ad libitum* (control, n = 22); (ii) 30% CR diet (CR, n = 22); (iii) control diet with leucine supplementation (5% of diet, w/w) consumed *ad libitum* (control + LEU, n = 22); or (iv) 30% CR diet with leucine supplementation (CR + LEU, n = 22). The AIN-76A control diet, when consumed *ad libitum*, results in an overweight phenotype characterized by steady weight gain, while the CR results in a lean phenotype characterized by weight maintenance [28,29]. Both CR diets were administered as a daily aliquot providing 70% of the total energy but 100% of the vitamins, minerals, amino acids and fatty acids consumed by the controls. Leucine was purchased from AIDP, Inc. (City of Industry, CA, USA) and was incorporated into the AIN-76A diet premix to provide 50 g/kg feed, or 5% dietary leucine supplementation. This dose of leucine is commonly used in animal studies of muscle regeneration [30,31]. All diets were purchased from Research Diets (New Brunswick, NJ, USA).

Energy intakes and body weights for each mouse were recorded weekly for 21 weeks, and then glucose tolerance tests (GTTs) were performed on a randomly selected subset of animals (n = 10/group). After injecting a 20% (w/v) glucose solution, blood glucose levels were measured with a Contour glucometer (Bayer HealthCare LLC, Mishawaka, IN, USA) at baseline, 15, 30, 60, and 120 minutes. Also after the rats were on the diet for 21 weeks, quantitative magnetic resonance (qMR) analysis (EchoMRI, Houston, TX) was done on a randomly selected subset of animals (n = 10/group) to obtain percent body fat and lean mass. At 22 weeks on the diet, all the mice were fasted for 12 hours and blood samples were collected from the retro-orbital venous plexus. After coagulating at room temperature for 30 minutes, blood samples were centrifuged at 9,300 × g for 5 minutes. Serum was separated, then snap-frozen and stored at −80°C until assayed for hormones. At 23 weeks on the diet, randomly selected mice (control, n = 7; control + LEU, n = 6; CR, n = 6; and CR + LEU, n = 7) were fasted for 12 hours and anesthetized by CO_2_ inhalation. Blood was collected by cardiac puncture, and pancreata were collected and stored for analyses other than those outlined in this manuscript. One mouse from the control + LEU group died, and one mouse from the CR group died before week 23. All remaining mice (n = 15/group) were subcutaneously injected into the right flank with 500,000 Panc02 cells (kindly provided by Dr. J. Schlom, National Cancer Institute, Bethesda, MD, USA) suspended in serum-free McCoy’s 5A medium. Once palpable, tumors were measured weekly with calipers, and tumor volume was approximated using the formula for an ellipsoid (4/3πr_1_^2^r_2_).

At 27 weeks on the diet, mice were fasted for 12 hours and then anesthetized by CO_2_ inhalation. They then underwent cardiac puncture for blood collection and were subsequently killed by cervical dislocation. Pancreatic tumors were harvested and either snap-frozen in liquid nitrogen and stored at −80°C, or fixed with 10% neutral-buffered formalin overnight, switched to 70% ethanol, paraffin embedded, subsequently used for immunohistochemical analyses.

### Serum hormones

After study termination, serum insulin and leptin levels were analyzed using Lincoplex™ bead-based multiplexed assays (Millipore, Billerica, MA, USA; MADPK-71 K-07). Serum adiponectin and IGF-1 were quantified by singleplex assay kits (Millipore; MADPK-71 K-ADPN and RMIGF187K, respectively). All assays were analyzed using a BioRad Bioplex™ analyzer (BioRad, Hercules, CA, USA) according to manufacturer’s directions.

### Immunohistochemical analyses

Formalin-fixed tissues were embedded in paraffin, cut into 4-μm thick sections, and processed for immunohistochemistry at the Histology Core Laboratory at The U.T. M.D. Anderson Cancer Center, Science Park Research Division (Smithville, TX, USA). Antibodies used for immunohistochemistry were optimized by core personnel using positive and negative controls for each analysis. Slides were deparaffinized in xylene and sequentially rehydrated in ethanol to water. Endogenous peroxidase activity was quenched with 3% hydrogen peroxide for 10 minutes. Antigen retrieval required microwaving slides with 10 mM citrate buffer. Nonspecific binding was blocked by treating sections with Biocare blocking reagent (Biocare Medical, Concord, CA, USA) for 30 minutes at room temperature, followed by incubation with primary antibody diluted in blocking buffer overnight at 4°C. The following primary antibodies and dilutions were used: phospho-S6 ribosomal protein_S235/236_ (Cell Signaling, Danvers, MA, USA; 1:100); phospho-mTOR_Ser2448_ (Cell Signaling; 1:50); phospho-ACC_Ser79_ (Cell Signaling; 1:50); Ki-67 (Dako, Carpinteria, CA, USA; 1:200); cyclin D1 (Santa Cruz Biotechnology, Santa Cruz, CA, USA; 1:500); and cleaved caspase-3 (R&D Systems, Minneapolis, MN, USA; 1:500). Slides were washed twice in PBS, incubated for 30 minutes with secondary antibody, washed two times with PBS, stained with diaminobenzidine (BioGenex, Fremont, CA, USA) and counterstained with hematoxylin. Images were captured by the Aperio ScanScope XT (Aperio Technologies, Vista, CA, USA) and staining was quantified using the Aperio ImageScope (Aperio Technologies). For Ki-67, phospho-mTOR, and cyclin D1 quantification, automated algorithms were used to determine negative or positive nuclear staining. The percentage of positive cells was obtained with 20× objective in pancreatic tumor sections. Positive staining was defined as 1+, 2+, and 3+ for cyclin D1 and phospho-ACC. Positive staining for phospho-mTOR was defined as only 2+ and 3+ due to its high baseline phosphorylation. Cleaved caspase-3 (CC3) was quantified as the average area of positively stained cells, with positive staining defined as at least 70% of a 100 μm × 100 μm section. For all proteins, the positive staining was averaged per group (n = 5/group).

### *In vitro* studies

Panc02 cells were cultured in a 37°C incubator under 5% CO_2_ with McCoy’s 5A media with glutamine (HyClone, Logan, UT, USA) and 3 g/L glucose but without BCAA, and then supplemented with penicillin/streptomycin, nonessential amino acids, sodium pyruvate, HEPES, 10% heat-inactivated FBS (HyClone), and physiological levels of leucine, isoleucine, and valine (MP Biomedicals, Santa Ana, CA, USA) [32]. For western blotting, approximately 100,000 Panc02 cells were seeded in 6-well plates and allowed to settle overnight in McCoy’s 5A media with 10% FBS. Cells were then treated with McCoy’s 5A plus 10% FBS with or without leucine supplementation and McCoy’s 5A plus 1% FBS with or without leucine supplementation (MP Biomedicals, Santa Ana, CA, USA). For western blot analysis, cells were treated for 20 min with 0.3 mM leucine after 3 hours of media pretreatment.

### Western blotting

Panc02 cells were lysed on ice for 1 hour in RIPA buffer (Sigma, St. Louis, MO, USA) with protease inhibitor tablet (Roche Applied Sciences, Indianapolis, IN, USA) and phosphatase inhibitor cocktails II and III (Sigma). Protein lysates (40 μg) were resolved by SDS-PAGE using 6%, transferred to PVDF membranes (Bio-Rad, Hercules, CA, USA) overnight at 25 volts and blocked for 1 hour at room temperature with LI-COR Blocking Buffer (LI-COR Biotechnologies, Lincoln, NE, USA). Membranes were incubated overnight at 4°C with primary antibody (from Cell Signaling unless otherwise stated) diluted in 5% BSA (Santa Cruz) and specific for: phospho-ACC_Ser79_ (1:1000), β-actin (1:10000; Sigma), phospho-AMPKα_T172_ (1:1000), cleaved caspase-3 (1:1000), phospho-mTOR_Ser2448_ (1:1000), phospho-p70S6K_T389_ (1:1000), and phospho-S6 ribosomal protein_Ser235/236_ (1:1000). β-actin was used as a loading control for all antibodies. After three washes (5 minutes each) in 0.1% Tween-20/PBS (PBS-T), membranes were incubated for 1 hour at room temperature in species-specific secondary antibody (LI-COR) diluted (1:5000) in LI-COR Blocking Buffer. Following two washes in PBS-T and one wash in PBS, membranes were scanned using the Odyssey infrared fluorescent imaging system (LI-COR). Densitometry was performed using LI-COR software (LI-COR). Relative levels of proteins were calculated from three biological replicates.

### Cell proliferation assay

Cell viability was measured by the 3-(4,5-dimethyl-2-thiazolyl)-2,5-diphenyl-2H-tetrazolium bromide (MTT) Cell Proliferation Assay Kit (Trevigen, Gaithersburg, MD; 4890-025-K). In 96-well plates, Panc02 (1500 cells/well) in media were allowed to adhere overnight. Each well was filled with fresh treatment media supplemented with different amounts of FBS (10% or 1%) and leucine (0 or 0.3 mM). The cells were then incubated for 24 h at 37°C, exposed to fresh treatment media after the removal of old media, and incubated for an additional 24 h. MTT was added at a 1:10 ratio for 2 h, then the liquid was aspirated and 100 μL of dimethyl sulfoxide (DMSO) was added to lyse the cells and dissolve the solid residue. The optical density of each well at 570 nm and 690 nm, a reference wavelength, was determined using the Synergy 2 Multi-Detection Microplate Reader and Gen5 Data Analysis Software (Fisher Scientific, Pittsburgh, PA). Relative cell viability was then calculated using the absorbance of cells grown in media with 10% FBS and no leucine supplementation for normalization. Data shown represent the average of three biological replicates.

### Statistical analysis

Statistical analyses were conducted using GraphPad Prism (GraphPad Software, La Jolla, CA). Temporal differences between groups with respect to body weight and energy intake were assessed using repeated measures analysis; final measurements were compared using one-way ANOVA and Newman-Keul’s post hoc test of significance. Blood glucose levels at each time point were compared using one-way ANOVA followed by Newman-Keul’s post hoc test of significance, and overall blood glucose differences were compared by performing a one-way ANOVA on calculated areas under the curve followed by Newman-Keul’s post hoc test of significance. Pretumor serum hormone levels, percent body fat, lean mass, and fasting glucose at week 21 were compared by one-way ANOVA followed by Newman-Keul’s post hoc test of significance. Final measurements of *ex vivo* tumor volume and immunohistochemical staining of all antibodies were also compared among the groups by one-way ANOVA followed by Newman-Keul’s post hoc test of significance. To compare the effects of leucine supplementation in media with either 10% FBS or 1% FBS, western blot densitometry and relative cell viabilities at their respective time points were compared by two-tailed t-tests. Results were considered significant if *P* <0.05.

## Results

### Effects of calorie restriction and leucine supplementation on body composition, glucose homeostasis, and serum hormones

**Figure 1:**
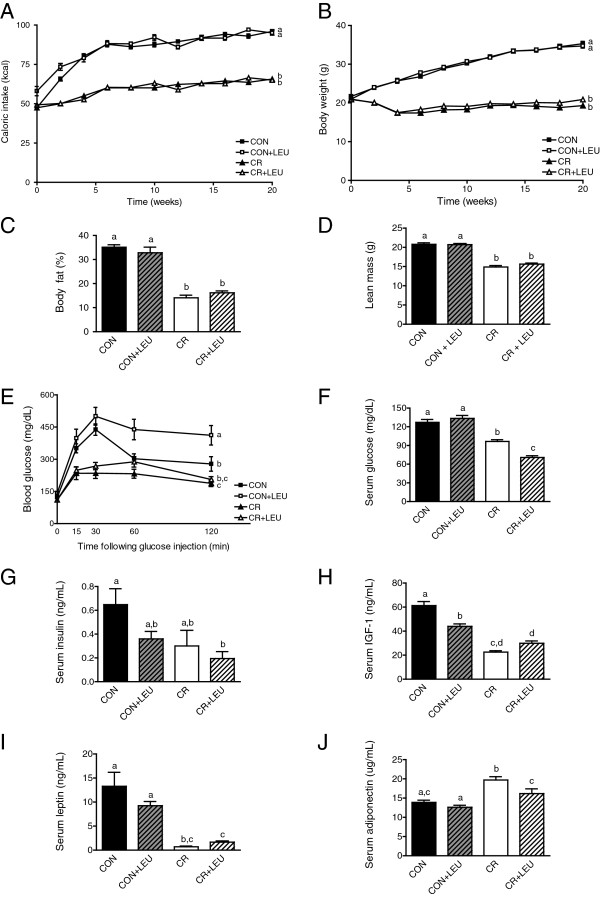
**Effects of calorie restriction (CR) and/or leucine (LEU) supplementation on body composition glucose tolerance, and hormones. (A)** Caloric intake and **(B)** body weight of C57BL/6 male mice on control and CR diets with and without leucine supplementation reported until glucose tolerance test (GTT) and quantitative magnetic resonance imaging (qMRI) performed (21 weeks, n = 22/group; *P* <0.001 between groups with different letters). **(C)** qMRI quantification of body fat and **(D)** lean mass between mice fed control or CR diets with and without leucine supplementation for 21 weeks (n = 10/group; *P* <0.001 between all groups with different letters). **(E)** GTT performed after 21 weeks on diet (n = 10/group; *P* <0.05 between control and CR, *P* <0.001 between all other groups with different letters). **(F)** Fasting glucose levels after 21 weeks on diet (prior to tumor injection; control, n = 14; all other groups, n = 15) (*P* <0.001 between all groups with different letters). **(G-J)** Serum hormone analyses after 21 weeks on diet (prior to tumor injection) of **(G)** insulin (*P* <0.05 between control and CR + LEU), (H) IGF-1 (*P* <0.001 between groups with different letters), **(I)** leptin (*P* <0.001 between control and both CR groups; *P* <0.01 between control + LEU and both CR groups), and **(J)** adiponectin (*P* <0.001 between CR and both control groups; *P* <0.05 between all other groups with different letters) (control groups, n = 9; CR groups, n = 10). All data are presented as the mean with error bars indicating the SD **(A,B)** or SEM **(C-J)**. Differences are considered significant if *P* <0.05. Abbreviations: CON, control diet; CR,calorie restriction diet; LEU, leucine-supplemented diet.

Male C57BL/6 mice were fed a control diet with or without leucine supplementation, or a 30% CR diet with or without leucine supplementation, for 27 weeks (including 21 weeks of diet before GTTs and qMRs were performed). Relative to the control mice, the CR mice had significantly reduced caloric intake (n = 22/group; *P* <0.001), body weight (n = 22/group; *P* <0.001), body fat (n = 10/group; *P* <0.001), and lean mass (n = 10/group; *P* <0.001), irrespective of leucine supplementation (Figure 1A-D).

At 21 weeks of study, the CR group without leucine supplementation, relative to controls without leucine supplementation, displayed enhanced glucose clearance as assessed by GTT (n = 10/group; *P* <0.05), with blood glucose concentrations peaking in 15 minutes in CR mice and 30 minutes in control mice following glucose bolus (Figure 1E). Leucine supplementation significantly decreased glucose clearance in the context of the high-calorie control diet (n = 10/group; *P* <0.001), but did not significantly alter glucose uptake in the context of the CR diet (n = 10/group; *P* >0.05) (Figure 1E). Even at 6 weeks, leucine supplementation showed the same trend of inhibiting glucose clearance in mice on the control diet (see Additional file 1). The CR diet group without leucine supplementation showed significantly lower fasting serum glucose levels relative to the controls (control group, n = 14; CR group, n = 15; *P* <0.001) (Figure 1F), and leucine supplementation further reduced glucose levels in the CR mice (n = 15/group; *P* <0.001), but did not affect glucose levels in control mice (control group, n = 14; control group with leucine supplementation, n = 15; *P* >0.05).

The CR mice without leucine supplementation, relative to the control mice without leucine supplementation, had significantly lower serum levels of IGF-1 (control group, n = 9; CR group, n = 10; *P* <0.001) (Figure 1H) and leptin (control group, n = 9; CR group, n = 10; *P* <0.001) (Figure 1I) and higher levels of adiponectin (control group, n = 9; CR group, n = 10; *P* <0.001) (Figure 1J) but did not have significantly altered levels of insulin (control group, n = 9; CR group, n = 10; in *P* >0.05) (Figure 1G). Leucine supplementation lowered IGF-1 in the control group (n = 9/group; *P* <0.001) and reduced adiponectin (n = 10/group; *P* <0.05) in the CR mice, but caused no other alterations to the levels of the other energy-responsive serum hormones measured in either diet group (Figure 1G-H).

### Effects of calorie restriction and/or leucine supplementation on Panc02 tumor growth and apoptosis

**Figure 2:**
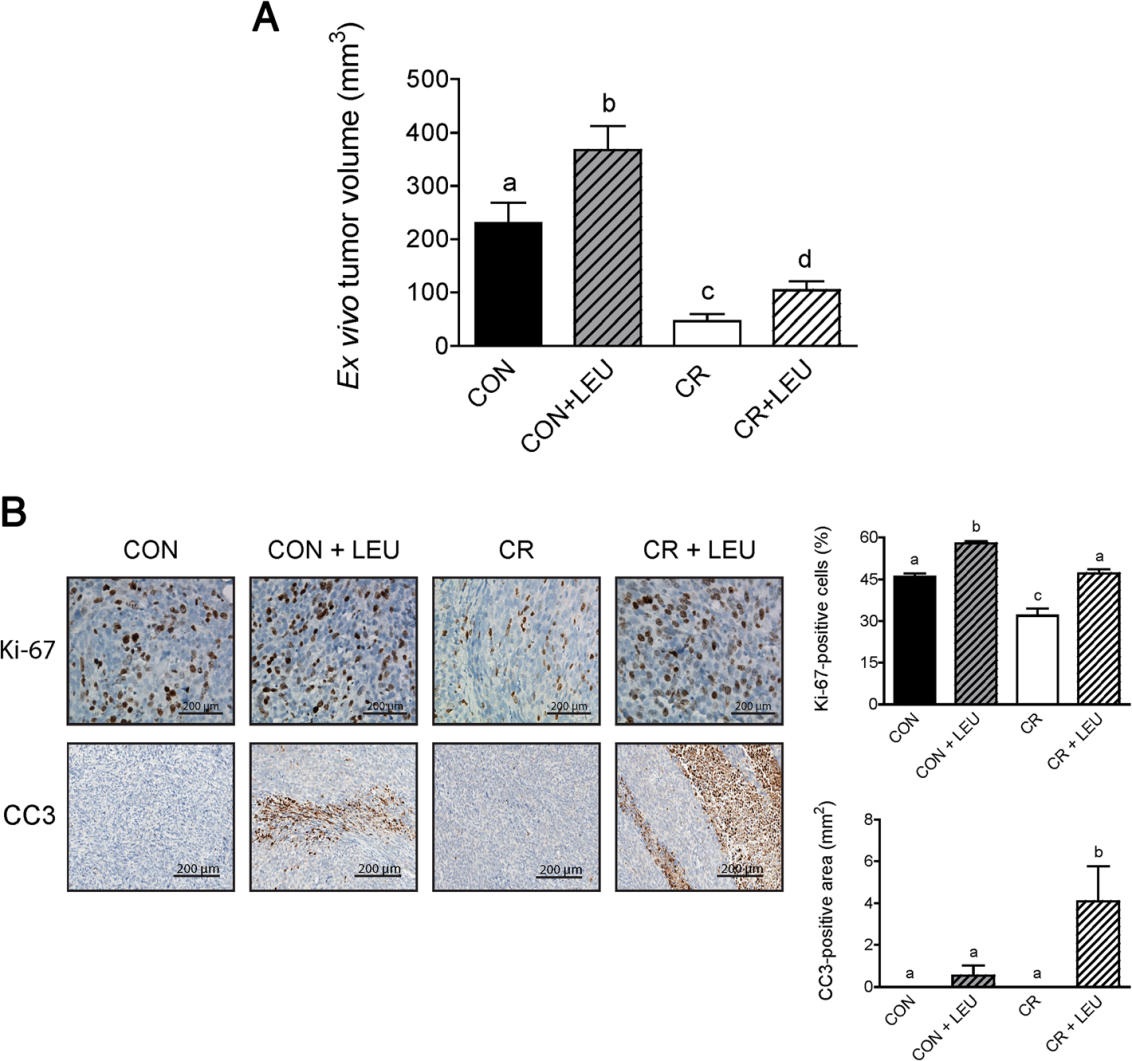
**Effects of leucine supplementation on Panc02 tumor growth and apoptosis. (A)** Differences in tumor volume between mice on control and calorie restriction (CR) diets with and without leucine (LEU) supplementation 4 weeks after tumor cell injections (control, control + LEU, and CR, n = 14/group; and CR + LEU, n = 13) (*P* <0.01 between control and both leucine-supplemented groups; *P* <0.001 between all other groups with different letters). **(B)** Comparison of immunohistochemical analyses performed on tumor sections for Ki-67 (n = 5/group; *P* <0.001 between all groups with different letters) and cleaved-caspase 3 (CC3) (n = 5/group; *P* <0.01 between the two leucine-supplemented groups; *P* <0.05 between all other groups with different letters). Scale bars represent 200 μm. Tumor volume is presented as mean ± SD, and Ki-67 and CC3 data are presented as mean ± SEM. Differences are considered significant if *P* <0.05. Abbreviations: CON, control diet; CR,calorie restriction diet; LEU, leucine-supplemented diet.

To interrogate whether leucine supplementation modulates murine pancreatic cancer cell growth in control mice, and/or impacts the anticancer response to CR, we injected mice from each diet group with Panc02 cells at week 23 and monitored tumor growth during the next 4 weeks. The final mean *ex vivo* tumor volume from CR mice, both with and without leucine supplementation, was significantly smaller than control mice (n = 14/group; *P* <0.001). However, leucine supplementation resulted in significantly larger tumors in both the control and CR diet groups, relative to each diet’s respective nonsupplemented group (control group and control with leucine supplementation group, n = 14; *P* <0.01) (CR group, n = 14; CR group with leucine supplementation, n = 13; *P* <0.001) (Figure 2A).

The influence of energy balance and leucine supplementation on cell proliferation was assessed in tumor tissues by immunohistochemical staining against Ki-67 (Figure 2B). While CR significantly reduced cell proliferation, relative to control diet, in nonsupplemented mice (n = 5/group; *P* <0.001), leucine supplementation significantly increased cell proliferation relative to the respective nonsupplemented mice within both diet groups (n = 5/group; *P* <0.001). The amount of Ki-67 staining in the leucine-supplemented CR group was augmented to the level of the nonsupplemented control group (n = 5/group; *P* <0.001).

Leucine supplementation in the CR group enhanced tumor proliferation more than it did tumor burden (Figure 2A,B), suggesting that final tumor size was influenced by both proliferation and apoptosis. Based on immunohistochemical analysis of tumors, we found no appreciable levels of CC3 in tumors from mice not supplemented with leucine; however, leucine supplementation in both the control and CR diet groups resulted in marked CC3-positive areas (n = 5/group; *P* <0.05) (Figure 2B). Leucine supplementation in the CR group resulted in much higher levels of apoptosis with 9.8 percent of the tumor composed of apoptotic areas in the CR group compared to 1.6 percent in the control group (Figure 2B). This increase in apoptosis could explain why leucine supplementation in the CR group, despite an equivalent level of proliferation as the control group, resulted in restrained tumor growth. Although apoptosis occurred in tumors of mice that consumed leucine-supplemented diets, 0.3 mM leucine supplementation *in vitro* did not significantly affect CC3 levels (Additional file 2) due to 1% FBS only partially modeling CR through growth factor reduction.

### CR and leucine supplementation have differential effects on energy responsive signaling intermediates

**Figure 3:**
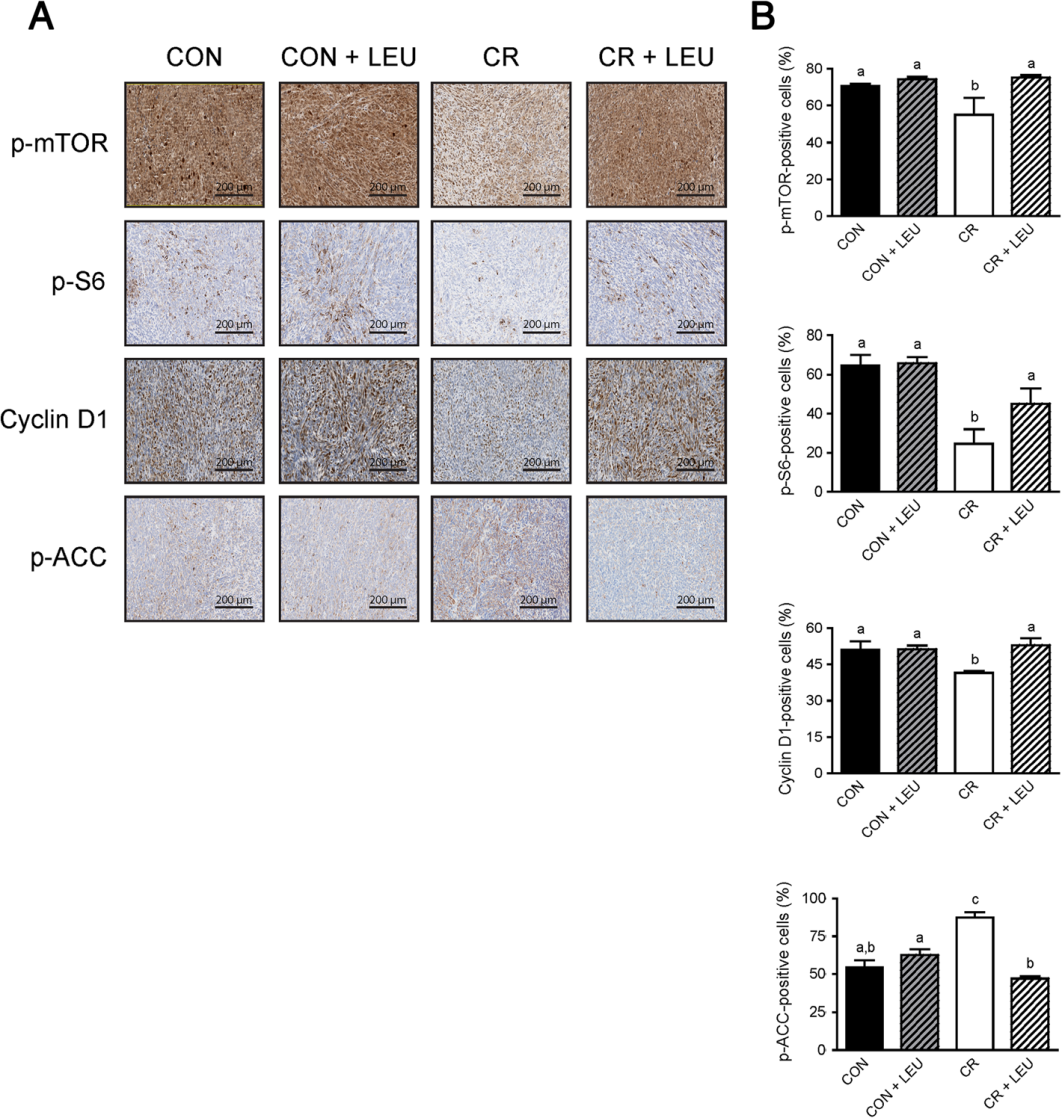
**Effects of calorie restriction (CR) and/or leucine (LEU) supplementation on energy responsive signals in Panc02 tumors. (A)** Comparison of immunohistochemical analyses on tumor sections for phospho-mTOR (*P* <0.05 between groups with different letters), phospho-S6 (*P* <0.001 between control and CR; *P* <0.01 between control + LEU and CR; *P* <0.05 between CR and CR + LEU), cyclin D1 (*P* <0.05 between groups with different letters), phospho-ACC (*P* <0.05 between the two leucine-supplemented groups; *P* <0.001 between all other groups with different letters) (n = 5/group). Scale bars represent 200 μm. All data are presented as the mean ± SEM. Differences are considered significant if *P* <0.05. Abbreviations: CON, control diet; CR,calorie restriction diet; LEU, leucine-supplemented diet.

The effects of energy balance and leucine supplementation on mTOR signaling were assessed by immunohistochemical analyses of the levels of phospho (p)-mTOR, p-ACC (a marker of AMPK activity, an upstream inhibitor of mTOR), and p-S6 and cyclin D1 (both downstream mTOR targets). Based on this analysis, we found that tumors from CR mice without leucine supplementation, relative to tumors from control mice without leucine supplementation, displayed increased levels of p-ACC (n = 5/group; *P* <0.05) and reduced levels of p-mTOR (n = 5/group; *P* <0.05) and its downstream effector, p-S6 ribosomal protein (n = 5/group; *P* <0.001). Additionally, tumors from CR mice, relative to tumors from control mice, showed significantly reduced levels of cyclin D1 (n = 5/group; *P* <0.05) (Figure 3A).

Leucine supplementation in the control diet did not significantly alter amounts of these energy responsive intermediates. However, leucine supplementation in the CR diet significantly reduced p-ACC (n = 5/group; *P* <0.001) and increased p-mTOR (n = 5/group; *P* <0.05), p-S6 (n = 5/group; *P* <0.05), and cyclin D1 (n = 5/group; *P* <0.05) to levels comparable to the nonsupplemented control group (Figure 3A-B).

### Effect of leucine supplementation on Panc02 cell lines

**Figure 4:**
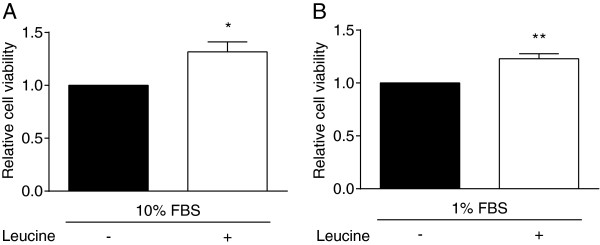
**Effects of leucine supplementation on viability of Panc02 tumor cells. (A-B)** Comparison of relative viability of cells grown in media with either **(A)** 10% fetal bovine serum (FBS) or **(B)** 1% FBS as assessed by MTT assays after 48 hours of 0.3 mM leucine supplementation (* = *P* <0.05, ** = *P* <0.01). All data are presented as the mean ± SEM. Differences are considered significant if *P* <0.05.

To confirm the proliferative effect of leucine supplementation seen *in vivo*, *in vitro* analyses were performed using the Panc02 cell line. To model the growth factor restrictive environment in CR mice relative to the overweight control mice as seen in Figure 1H, we grew the cells in media with either 1% FBS or 10% FBS. Supplementing media with 1% FBS has been used to mimic serum growth factor reduction found in calorie-restricted mice [33]. In the growth factor-rich environment of media with 10% FBS, cell viability was significantly increased by ~30% with 0.3 mM leucine supplementation (*P* <0.05) (Figure 4A). Leucine supplementation also increased cell viability by ~30% in the growth factor-restricted environment of media with 1% FBS (*P* <0.01) (Figure 4B). These 30% increases are similar to the increases in Ki-67 seen when comparing mice on leucine-supplemented diets to their respective nonsupplemented controls (Figure 2B). This 0.3 mM concentration of leucine were chosen based on experiments showing that: i) serum leucine increased by 0.3 mM in mice consuming a leucine-supplemented diet [34]; and ii) cell viability of Panc02 cells significantly increased with 0.3 mM leucine supplementation *in vitro* (Additional file 3).

### Effect of leucine supplementation on mTOR pathway intermediates

**Figure 5:**
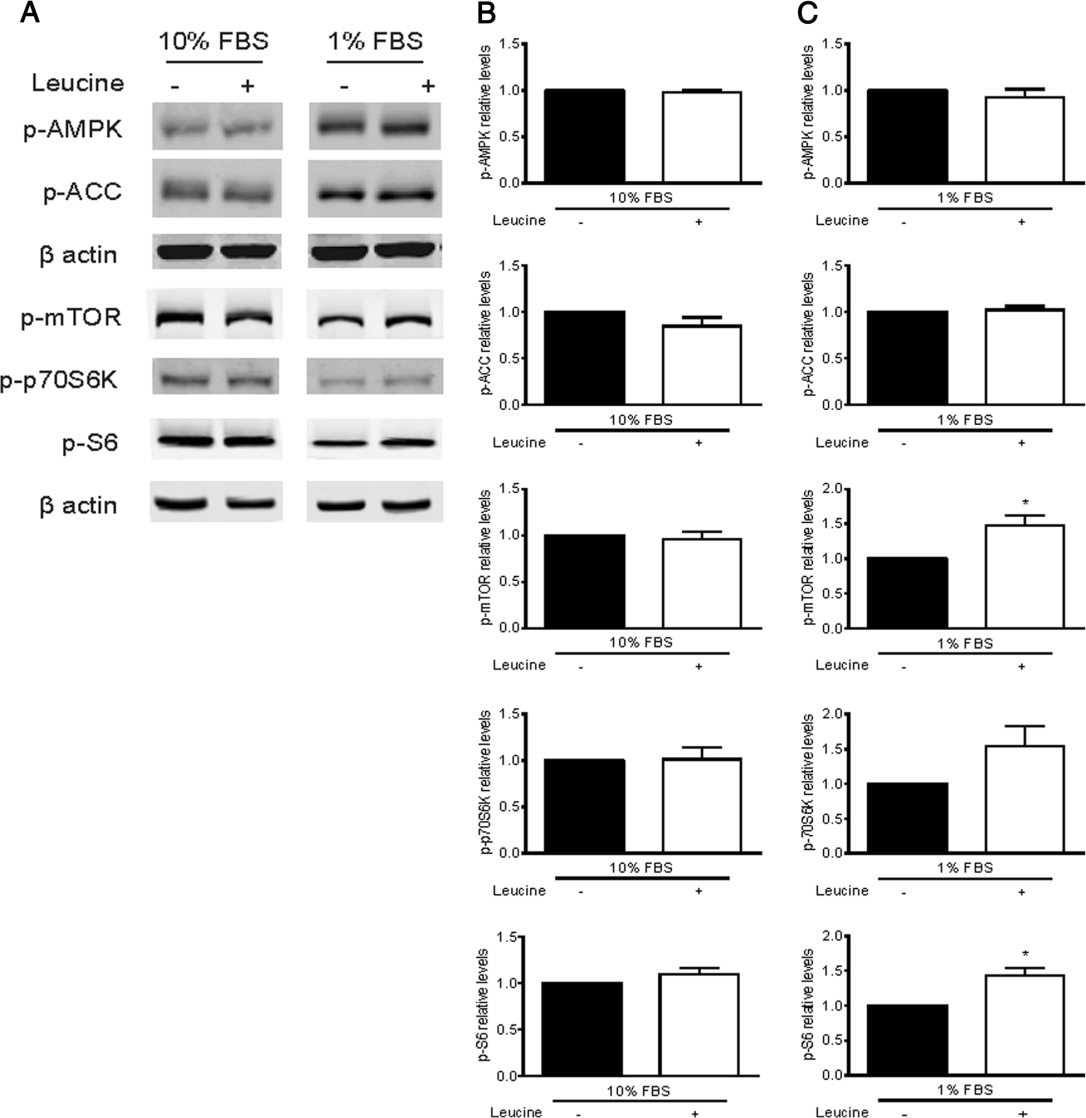
**Effects of leucine supplementation on energy responsive signals of Panc02 tumor cells. (A-C)** Western blot analysis of phosphorylated AMPK, ACC, mTOR, p70S6K, and S6 after 20 minutes of 0.3 mM leucine administration after pretreatment with respective media for 3 hours. Data shown are representative blots from three biological replicates, and images for each protein are from the same blot. **(B-C)** Relative phosphorylation of p-AMPK, p-ACC, p-mTOR, p-p70S6K, and p-S6 in cells grown in media with either **(A)** 10% FBS or **(B)** 1% FBS with or without leucine supplementation (* = *P* <0.05). All data are presented as the mean ± SEM. Differences are considered significant if *P* <0.05.

In order to understand the differential response to leucine supplementation between the diet groups with respect to mTOR signaling, *in vitro* analyses were performed using Panc02 cell lines. Western blot analyses for the energy responsive intermediates p-AMPK, p-ACC, p-mTOR, p-p70S6K, and p-S6 revealed that the effects of leucine supplementation on cell signaling intermediates were impacted by growth factor availability. In the growth factor-rich environment of media with 10% FBS, supplementation with 0.3 mM leucine had no effect on phosphorylated AMPK, ACC, mTOR, p70S6K, or S6 ribosomal protein (Figure 5A,B). In the 1% FBS setting, leucine supplementation had no effect on phosphorylated AMPK or ACC, but did significantly increase phosphorylated mTOR (*P* <0.05) and its downstream effector S6 ribosomal protein (*P* <0.05). Another downstream effector of mTOR, p-p70S6K, was also increased with leucine supplementation in the 1% FBS setting, although the difference was not statistically significant (Figure 5A,C).

## Discussion

Findings in this report demonstrate for the first time that dietary leucine supplementation increases growth of pancreatic tumors. More specifically, we show that leucine supplementation not only enhances the protumorigenic nature of a high calorie, high carbohydrate control diet, but also partially overcomes the well-established anticancer effects of CR. The mechanisms underlying these leucine-induced protumor effects may be diet-dependent, suggested by increased glucose availability in overweight mice and increased activation of the mTOR protein synthetic pathway in CR mice.

Mice administered the leucine-supplemented control diet developed the largest tumors and had the highest level of tumor cell proliferation of all four groups. The increased tumor burden observed in the leucine-supplemented control group (relative to controls without leucine supplementation) occurred without significant changes in tumoral apoptosis or mTOR activation, as evidenced by unchanged levels of both p-AMPK, an upstream inhibitor of mTOR, and p-S6, a downstream effector of mTOR. In overweight control mice, high basal levels of circulating IGF-1 and tumoral mTOR activity are consistently found [10]. This high level of activity likely blunted any further increase in mTOR activation in response to leucine supplementation in the control diet, suggesting a biological threshold was attained. This concept of a biological threshold for mTOR phosphorylation was substantiated using Panc02 cancer cells *in vitro*, because mTOR activation was only enhanced in response to leucine under growth factor restrictive conditions (1% FBS) and not growth factor-abundant conditions (10% FBS). The enhanced tumor growth in the leucine-supplemented control group cannot be explained by changes in mTOR signaling in the tumor, but was associated with greater glucose availability (reduced fasting insulin levels and diminished glucose clearance). High levels of glucose have been shown to increase proliferation in multiple pancreatic cancer cell lines by stimulating glucose consumption and metabolism [35,36]. Although the noted effects on insulin levels in the control group contradict the putative characteristics of leucine as an insulin secretagogue and enhancer of blood glucose disposal in patients with type 2 diabetes [37], recent evidence suggests that leucine’s effects on glucose sensitivity differ depending on physiologic context, i.e., diabetic versus non-diabetic state [38]. In a physiologic scenario, leucine stimulates mTOR activity in the β-cells of the pancreas and promotes proliferation and thus insulin secretion [38]. On the other hand, chronic β-cell hyperfunction, a consequence of excessive leucine exposure, results in accelerated β-cell apoptosis and eventual secretory deficiency through a negative feedback loop involving the mTOR-dependent inhibition of IRS-1 [39]. Indeed, a diet consisting of high levels of leucine combined with saturated fatty acids results in insulin resistance in rodents [40], and chronic infusion of amino acids at high concentrations induces insulin resistance in humans [41]. Leucine supplementation did not induce insulin resistance in mice on the CR regimen. CR has been shown to decrease basal p70S6K activation, which may have protected against mTOR-dependent β-cell hyperfunction [40]. Taken together, our data suggest that control tumors obtained a leucine-induced growth advantage because of increased glucose availability as a consequence of either impaired insulin secretion or function.

Mice administered the CR diet without leucine supplementation had the smallest tumors and lowest level of tumor cell proliferation, while mice fed the leucine-supplemented CR regimen (relative to CR mice without leucine supplementation) had increased tumor growth to levels intermediate between the unsupplemented mice on the CR and control diets. Leucine supplementation in the CR diet, relative to CR alone, also increased tumor cell proliferation (to the levels observed in control mice), and increased apoptosis. It is not uncommon to observe increases in both cell proliferation and cell death in the same tumor, as seen in the tumors of mice on the CR diet. In fact, a number of dominant oncogenes that increase proliferation through induction of aberrant growth signals, also induce apoptosis [42]. Thus, leucine-induced dysregulation of growth signals, such as mTOR activation, in a setting of low-energy substrates and growth factors in response to a CR regimen, may explain the observed increases in apoptosis, tumor cell proliferation (to control levels), and partial rescue of tumor burden in the leucine-supplemented CR mice. This rescue of tumor burden was only partial, because leucine significantly increased proliferation. As previously stated, high levels of mTOR activity support proliferation and survival of pancreatic cancer cells, and CR consistently results in decreased activation of mTOR in pancreatic tumors [13]. In contrast to tumors from the leucine-supplemented control group, we found that tumors from the leucine-supplemented CR group demonstrated marked increases in mTOR activation, as evidenced by lower levels of p-AMPK and higher levels of p-S6 and cyclin D1, without changes in fasting insulin levels and glucose clearance. The maintenance of physiologic insulin secretion in the CR mice was perhaps due to the protection of β-cells by chronic CR, a strategy that has been shown to increase β-cell proliferation in rats [43]. Taken together, our data suggest that CR tumors obtained a leucine-induced growth advantage because of increased mTOR activation.

## Conclusions

This report establishes that dietary leucine supplementation, irrespective of energy balance status, promotes pancreatic tumor growth. These findings suggest caution regarding the clinical use of leucine supplementation for purposes of lean muscle enhancement in cachectic cancer patients. Additional research is needed to ascertain the impact amino acids (BC or otherwise) have on cancer growth and muscle repair; the identification of mTOR-independent approaches to spare muscle in cachectic cancer patients; and the link between energy balance, mTOR signaling, and amino acid metabolism.

## Abbreviations

ACC: acetyl-CoA carboxylase; AIN: American Institute of Nutrition; AMPK: AMP-activated protein kinase; BCAA: branched-chain amino acid; BSA: bovine serum albumin; CC3: cleaved caspase-3; CR: calorie restriction; FBS: fetal bovine serum; GTT: glucose tolerance test; IGF-1: insulin-like growth factor-1; IRS-1: insulin receptor substrate-1; mTOR: mammalian target of rapamycin; p70S6K: p70S6 kinase; PBS: phosphate-buffered saline; PBS-T: phosphate-buffered saline with tween; PI: propidium iodide; PVDF: polyvinylidene difluoride; qMRI: quantitative magnetic resonance imaging; RIPA: radioimmunoprecipitation assay; SDS-PAGE: sodium dodecyl sulphate-polyacrylamide gel electrophoresis.

## Competing interests

The authors declare that they have no conflicts or competing interests.

## Authors’ contributions

KAL conducted *in vitro* analysis, including western blot analysis and DNA labeling, analyzed IHC staining of tumor tissue, performed some of the statistical analysis, and drafted the manuscript. LML participated in study design, performed some of the animal techniques, analyzed a portion of the IHC data, and edited the manuscript. AJR designed and conducted the animal study, performed some of the statistical analysis, and edited the manuscript. SDH designed the study and edited the manuscript. All authors read and approved the final manuscript.

## Supplementary Material

Additional file 1 **Effects of leucine supplementation on glucose tolerance at 6 weeks.** Glucose tolerance test (GTT) performed after 6 weeks on diet (n = 10/group; *P* <0.001 between control with leucine supplementation and the calorie restriction (CR) groups; *P* <0.05 between the control groups). All data are presented as the mean ± SEM. Differences are considered significant if *P* <0.05. Click here for file

Additional file 2 **Effects of single BCAA supplementation on apoptosis of Panc02 tumor cells.** Western blot analysis of cleaved caspase-3 protein levels after 24 hours of either 0.3 mM leucine, isoleucine, or valine administration. Data shown are representative blots from three biological replicates. Relative protein levels of cleaved caspase-3 were quantified by densitometry using LI-COR Odyssey software. All data are presented as the mean ± SEM. Differences are considered significant if *P* <0.05. Click here for file

Additional file 3 **Effects of different doses of leucine supplementation on Panc02 tumor cell viability.** Comparison of relative cell viability as assessed by MTT assays after 48 hours of leucine supplementation (* = *P* <0.05). All data are presented as the mean ± SEM. Differences are considered significant if *P* <0.05. Click here for file
