# Supplementary figures and images for: Leucine supplementation differentially enhances pancreatic cancer growth in lean and overweight mice
Source: Cancer Metab. 2014 Mar 31;2:6. doi: 10.1186/2049-3002-2-6 (PMC4392529; doi:10.1186/2049-3002-2-6)

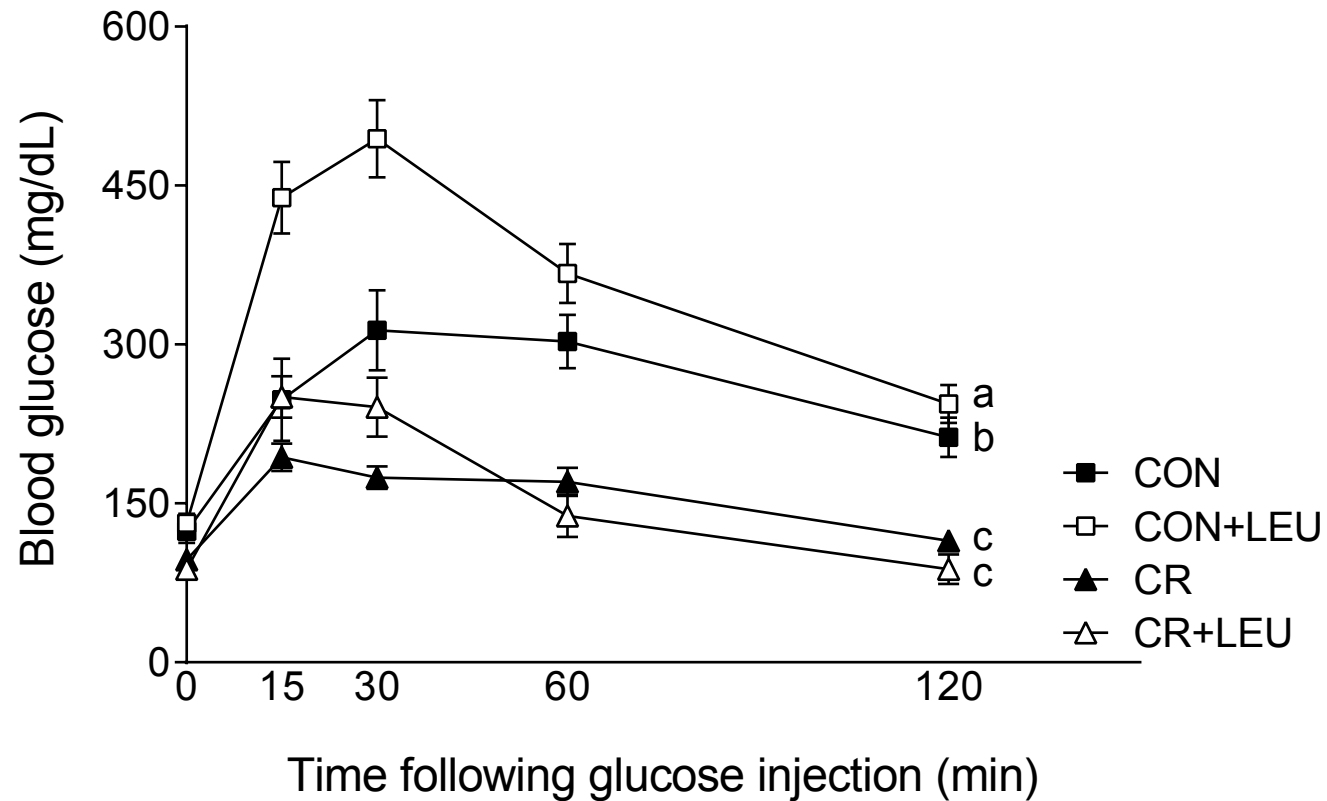

Supplement: Additional file 1 — Effects of leucine supplementation on glucose tolerance at 6 weeks. Glucose tolerance test (GTT) performed after 6 weeks on diet (n = 10/group; P <0.001 between control with leucine supplementation and the calorie restriction (CR) groups; P <0.05 between the control groups). All data are presented as the mean ± SEM. Differences are considered significant if P <0.05. [file 2049-3002-2-6-S1.pdf]

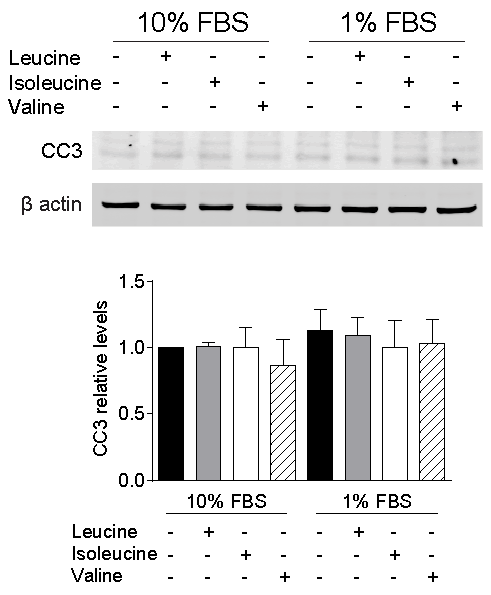

Supplement: Additional file 2 — Effects of single BCAA supplementation on apoptosis of Panc02 tumor cells. Western blot analysis of cleaved caspase-3 protein levels after 24 hours of either 0.3 mM leucine, isoleucine, or valine administration. Data shown are representative blots from three biological replicates. Relative protein levels of cleaved caspase-3 were quantified by densitometry using LI-COR Odyssey software. All data are presented as the mean ± SEM. Differences are considered significant if P <0.05. [file 2049-3002-2-6-S2.tiff]

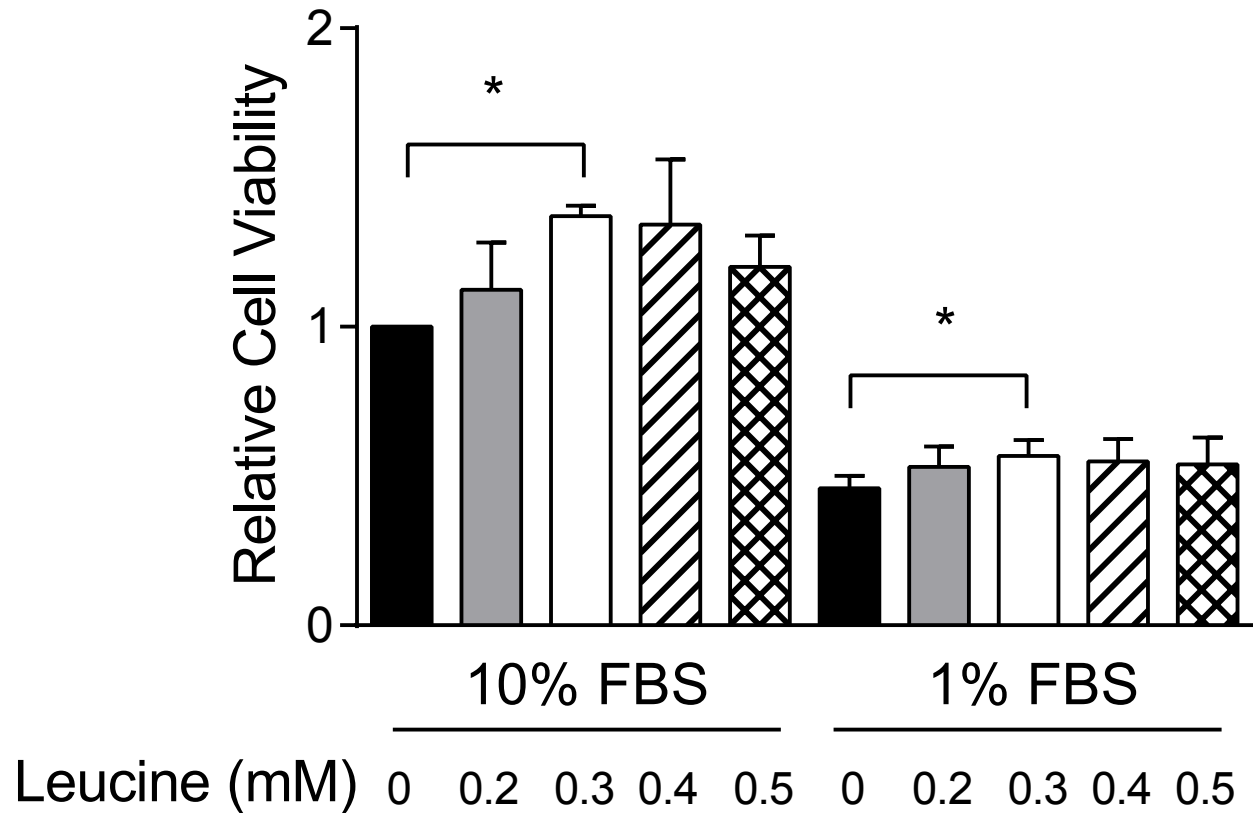

Supplement: Additional file 3 — Effects of different doses of leucine supplementation on Panc02 tumor cell viability. Comparison of relative cell viability as assessed by MTT assays after 48 hours of leucine supplementation (* = P <0.05). All data are presented as the mean ± SEM. Differences are considered significant if P <0.05. [file 2049-3002-2-6-S3.pdf]
